# Endoscopic Ultrasound-Guided Fine-Needle Biopsy versus Fine-Needle Aspiration in the Diagnosis of Focal Liver Lesions: Prospective Head-to-Head Comparison

**DOI:** 10.3390/diagnostics12092214

**Published:** 2022-09-13

**Authors:** Marcel Gheorghiu, Andrada Seicean, Sorana D. Bolboacă, Ioana Rusu, Radu Seicean, Cristina Pojoga, Ofelia Moșteanu, Zeno Sparchez

**Affiliations:** 1 Department of Gastroenterology, Iuliu Hațieganu University of Medicine and Pharmacy, 400192 Cluj-Napoca, Romania; 2 Department of Gastroenterology, Regional Institute of Gastroenterology and Hepatology Prof. Dr. Octavian Fodor, 400192 Cluj-Napoca, Romania; 3 Department of Medical Informatics and Biostatistics, Iuliu Hațieganu University of Medicine and Pharmacy, 400349 Cluj-Napoca, Romania; 4 First Surgical Department, Hațieganu University of Medicine and Pharmacy, 400005 Cluj-Napoca, Romania; 5 Department of Clinical Psychology and Psychotherapy, Babeș-Bolyai University, 400029 Cluj-Napoca, Romania

**Keywords:** fine-needle aspiration (FNA), fine-needle biopsy (FNB), focal liver lesions, endoscopic ultrasonography (EUS), diagnostic, immunohistochemistry, liver

## Abstract

Endoscopic ultrasound-guided fine-needle biopsy (EUS-FNB) or fine-needle aspiration (EUS-FNA) from focal liver lesions are indicated in selected cases, but there has been no previous comparison of needle types of the same size. The aim of our study was to compare the histologic diagnostic accuracy and adequacy of cores obtained with EUS-FNB needles in contrast to those obtained with FNA needles in focal liver lesions. This prospective one-center study included patients with left lobe hepatic focal lesions with contraindications for percutaneous liver biopsy or need for EUS for concomitant lesions. Each patient had one pass of 22G EUS-FNB (Franseen) needle and one pass of 22G EUS-FNA in a crossover manner, without macroscopic on-site evaluation. Each sample was analyzed separately for histologic adequacy and diagnosis. The final diagnosis was based on histology results or on imaging follow-up in the case of negative biopsies. The EUS-FNB samples (n = 30) were found to be more adequate for histologic analysis, with more cellularity and longer tissue aggregates than the EUS-FNA samples (n = 30). The accuracy of EUS-FNB was 100%, whereas that of EUS-FNA was 86.7% (*p* = 0.039). No post-procedure complications were noted. The 22G EUS-FNB needle proved superior to 22G EUS-FNA in terms of tissue acquisition diagnostic accuracy and histologic adequacy in focal liver lesions.

## 1. Introduction

Focal liver lesions require histological and immunohistochemical diagnosis for appropriate treatment, especially in the context of malignant disease. Endosonography (EUS) can visualize hepatic lesions <1 cm, which were not identified by cross-sectional imaging, especially during staging for digestive cancers, although it is limited to the left lobe and the proximal region of the right lobe [1].

Classically, liver biopsies are obtained percutaneously using ultrasonographic guidance, but this operation cannot be performed in the case of ascites or subcapsular or very deep lesions. In such situations, EUS-guided hepatic tissue acquisition may be indicated. Other possible indications include the biopsy of hepatic lesions (metastases) during staging of concomitant biliopancreatic or gastric tumors or if the sample obtained by the percutaneous route is not adequate for pathological evaluation [2].

The diagnostic performances of liver biopsies through EUS guidance were largely assessed for diffuse hepatic diseases, and their results are comparable to those of percutaneous liver biopsies [3]. The samples obtained by the EUS approach are longer but more fragmented compared to those obtained by the percutaneous route [3]. The diagnostic yield for diffuse hepatic diseases was reported as 95.8% for FNA (fine-needle aspiration) [4] and up to 100% for FNB (fine-needle biopsy) [5]. The total sampled specimen length was similar for EUS-FNB and EUS-FNA (51.9 vs. 48.9 mm), but the portal tract number was higher (18 vs. 10, *p* = 0.003) for EUS_FNB in a meta-analysis of 1326 patients [5]. This corresponds to the requirements of the AASLD (American Association for the Study of Liver Diseases) for an adequate specimen of liver biopsy with specimen length (>15–20 mm) and portal tract number (>11–20) [6], sustaining the appropriate use of the EUS approach for diffuse hepatic diseases. However, the superiority of FNA or FNB needles for diffuse diseases has not been clearly established to date [7,8].

In the case of focal liver lesions, initial studies using EUS-FNA needles showed a diagnostic accuracy of 86.3–86.7% [9,10]. The sample quality could not always offer sufficient material for immunohistochemistry (biopsy sufficiency rate = 86.3%) [9], and multiple passes of the needle were required [10], with an increased risk of side effects. The reported diagnostic accuracy of first-generation FNB needles (pro-core needles) is 85.7–90% [11,12,13]. A randomized study comparing first-generation FNB needles and FNA needles reported fewer passes for FNB needles (1 vs. 2) and similar histology core but with similar diagnostic accuracy (81.5% vs. 80%) [14], although this value was lower that reported in other studies [9,10,11,12,13].

A meta-analysis comprising 18 randomized control trials in pancreatic and non-pancreatic lesions revealed that FNB assured better accuracy compared to FNA (87% vs. 80%, *p* = 0.02), with fewer passes and higher tissue core rates (80% vs. 62%) [15]. To the best of our knowledge, no study to date has reported on 22G Franseen EUS-FNB and EUS-FNA needles for focal liver lesions.

The aim of our study was to compare the diagnostic value and adequacy of FNB and FNA samples in focal liver lesions. The primary outcome was the diagnostic accuracy of EUS-FNB compared to standard EUS-FNA needles for specific focal hepatic lesions. The secondary outcomes were the evaluation of the adequacy of specimens for immunohistochemical (IHC) staining and the safety of EUS-FNB needles versus the standard FNA needles.

## 2. Materials and Methods

A trial with prospective inclusion of patients was conducted from January 2019 to March 2021 at one tertiary medical center, the Regional Institute of Gastroenterology and Hepatology, with approval from the Hospital Institutional Review Board and the University Ethics Board (approval numbers 7070/2019 and 477/2019, respectively). All patients provided written informed consent in accordance with the Declaration of Helsinki.

### 2.1. Subjects and Data Collection

Consecutive patients (aged 18–90 years) diagnosed with solid hepatic masses by computed tomography (CT) scan and unsuitable for percutaneous liver biopsy or requiring a EUS-guided sampling from both pancreas and focal liver lesions were eligible for the study.

Exclusion criteria were: (1) participant refusal or contraindication of the proposed intervention; (2) platelet level < 50,000/mm^3^ and INR (international normalized ratio) > 1.5; (3) prior curative liver surgical treatment or chemoradiotherapy; (4) anesthetic risk ASA4 or contraindication for sedation.

### 2.2. Study Outcome and Definitions

The eligible patients who agreed to participate were analyzed by EUS, followed by one pass of EUS-FNB and a second pass of EUS-FNA in a crossover design. When any puncture failed or the visual core was <4 mm, a third standard EUS-FNA was performed and considered for the final diagnosis, as described by Iwashita et al. [16].

In cases in which the histological analysis was noncontributory, a second puncture with EUS-FNB was scheduled within one month after the index EUS-FNA.

The final diagnosis was based on EUS-FNB or EUS-FNA results or on repeated EUS-FNB results and follow-up imaging in cases of negative FNB/FNA for malignancy (CT at three months and subsequent transabdominal ultrasound at 3-month intervals for 12 months).

### 2.3. Procedure

All interventions were performed using a therapeutic linear array echoendoscope (GF-UCT 180 AL5; Olympus, Tokyo, Japan) with an Aloka Prosound F75 ultrasound machine. All interventions were undertaken by three endoscopists (A.S., C.P., and O.M.) using EUS-FNA 22G needles (Expect; Boston Scientific, Marlborough, MA, USA) and EUS-FNB 22G needles (Acquire, Boston Scientific, Marlborough, MA, USA). Patients fasted prior to the procedure and were intravenously sedated with midazolam or propofol, according to the preference of patients and indication of the anesthesiologists.

**Figure 1:**
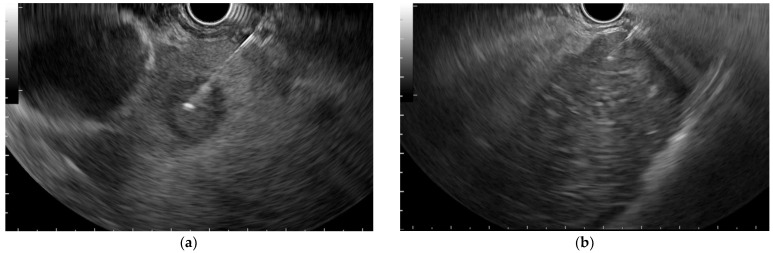
Endoscopic ultrasound tissue acquisition. (**a**) Hepatic metastasis of pancreatic adenocarcinoma; (**b**) hepatocarcinoma.

The EUS procedure included parenchyma analysis of the whole pancreas, left liver lobe, and right liver lobe in B mode EUS within the ultrasound-penetrating range. The first pass of EUS-FNB was performed using the no-fanning and the dry-suction techniques; the needle was moved back and forth ten times within the lesion while an assistant pulled the stylet and applied ten cc syringe suction (Figure 1). Then, EUS-FNA was performed in the same way as EUS-FNB in a crossover order.

After the procedure, the patients were supervised for 12 hrs. for adverse effects. The patients were recalled with the histological results (more than seven days), and adverse effects were noted. Procedure-related adverse events included bleeding, perforation, pneumothorax, bile leaks, and infections.

### 2.4. Preparation of Samples

No cytopathologist was present when the samples were collected, and no macroscopic on-site measurement was used in this study in order to maintain the comparison of one-to-one passes for each group [17,18]. The EUS-FNA and EUS-FNB visible cores were collected separately and inspected visually. The core was expelled by reintroducing the stylet into 10% buffered formalin. For histological analysis, the sample bottles were sent blinded (type of pass not identified).

The specimens were measured and paraffin-embedded, followed by staining with hematoxylin-eosin-safran with or without immunohistochemistry sections. One pathologist (I.R.) independently analyzed the samples, blinded to the sample provenance (EUS-FNA or EUS-FNB), but they had access to the clinical and imaging information.

A specimen was considered adequate for histologic examination if it contained a coherent tissue sample to make a diagnostic interpretation with recognizable structures of the targeted lesion. Positive specimens were categorized as unequivocally positive for malignancy. Specimens that contained inadequate material or atypia were not excluded from analysis but were considered negative for a diagnosis of malignancy (i.e., in an intention-to-diagnose analysis).

The histological result noted the length, cellularity, histologic core area, adequacy for diagnosis, appropriateness for immunohistochemistry, and the diagnosis for each sample. The length was measured microscopically after fixation, and the longest fragment core and the total fragment aggregate length were measured. Tumor cellularity was defined as the proportion of tumor cells in the specimen. Histologic core area was calculated by multiplying the total length of the cores by the thickness (ImageJ, https://imagej.nih.gov/ij/docs/pdfs/examples.pdf, accessed on 3 July 2022). Adequacy for diagnosis was defined as a sufficient amount of liver tissue to allow the pathologist to make a diagnostic interpretation, including immunohistochemistry (IHC). An adequacy score of 0 was considered insufficient for interpretation or sufficient material for interpretation; 3, limited histological interpretation; 4, adequate histological interpretation with low quality (<10× power field in length); and 5, adequate histological interpretation with high quality (>10× power field in length) [19]. The diagnosis was considered separately for each sample.

### 2.5. Statistics

The patient data were collected in a Microsoft Excel spreadsheet with the results of FNB and FNA as paired data (tumor cellularity, core tissue aggregate length (mm), longest core tissue length (mm), core surface (mm^2^), histology score, and adequate for IHC yield). Quantitative data were tested for distribution with Shapiro–Wilk test at a significance level. Data were reported as mean ± standard deviation and median (Q1 to Q3) where Q is the quartile and {min to max} to allow for a full view of the results. Comparisons between the investigated variables obtained by FNB and FNA were made with a two-tail Mann–Whitney test, and *p*-values < 0.05 were considered statistically significant. Qualitative variables were reported as absolute and relative frequencies. Sensitivity, specificity, positive predictive value, negative predictive value, accuracy, +CUI, and −CUI (where CUI is the clinical utility index) were used to evaluate the FNB and FNA performances. The performance metrics were reported with the associated 95% confidence intervals, expressed as [lower bound to upper bound] whenever appropriate. The performances of EUS-FNB and EUS-FNA were compared with Z-test for proportions whenever appropriate. The clinical utility index (CUI) was used to evaluate the relevance of FNB and FNA in screening and diagnosis (the higher the +CUI, the better for case finding; the higher the −CUI, the better for screening) [20].

## 3. Results

### 3.1. Participant Characteristics

Thirty-two consecutive patients were evaluated between January 2019 and March 2021. Two patients were excluded because the FNA and FNB samples were not analyzed separately following the study’s protocol. The characteristics of the evaluated patients are presented in Table 1.

The final diagnosis was based on EUS-FNB or EUS-FNA results in 25 patients with malignant lesions (14 metastases and 11 primary liver tumors). The remainder of the six benign lesions were abscesses with a solid aspect treated with antibiotics and were not further identified at the follow-up transabdominal imaging examination in all but one case with primary sclerosing cholangitis on MRI (magnetic resonance imaging) and repeated negative biopsy at 6-month follow-up.

### 3.2. B-Mode EUS Assessment

All focal liver lesions were hypoechoic or hyperechoic with a hypoechoic rim in the case of hepatic metastasis.

**Figure 2:**
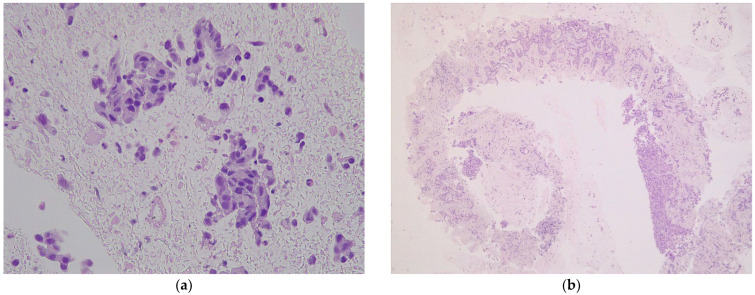
Microscopic aspects of endoscopic ultrasound specimen from an intrahepatic cholangiocarcinoma (hematoxylin-eosin): (**a**) FNA specimen (×40) with free-floating pleomorphic biliary epithelium without stromal support, rare neutrophils, and red blood cells; (**b**) FNB specimen (×5) with neoplastic biliary epithelium infiltrating cores of liver parenchyma.

**Figure 3:**
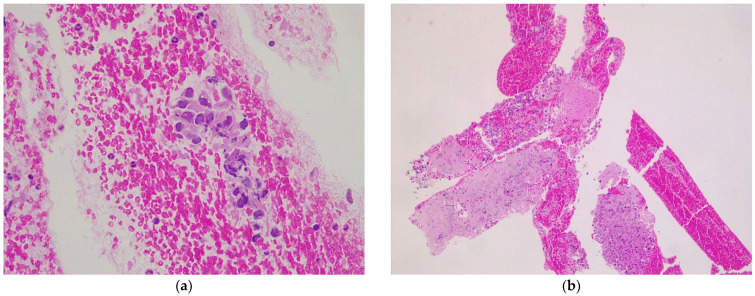
Microscopic aspects of endoscopic ultrasound specimen from a hepatic metastasis of pancreatic ductal adenocarcinoma (hematoxylin-eosin): (**a**) FNA specimen (×40) with free-floating pleomorphic glandular epithelium without stromal support and red blood cells; (**b**) FNB specimen (×5) with neoplastic glands infiltrating cores of liver parenchyma.

The adequacy of EUS-FNB compared to EUS-FNA samples is reported in Table 2. FNB samples were longer, larger, and more suitable for histology and IHC (Figure 2 and Figure 3). The median standardized histology score per pass was 5 (enough for adequate histology) in the EUS-FNB group for all lesions and 4 in the EUS-FNA group. No fragment was obtained in one EUS-FNB (benign as pathology) following the study’s protocol and in six cases of the EUS-FNA group (two benign and four malignant).

### 3.3. Diagnostic Yields in EUS-FNA Compared to EUS-FNA

EUS-FNB was found to provide a better diagnostic rate than EUS-FNA (Table 3), with excellent performances for case finding. The sensitivity and negative predictive value were better in the EUS-FNB group compared to EUS-FNA group. The diagnostic value expressed by clinical utility index (CUI) was excellent for diagnosis in both passes, and the CUI for screening was excellent in EUS-FNB passes and fair in the case of EUS-FNA passes.

## 4. Discussion

Our prospective study shows, for the first time, 22G FNB needles are statistical superior in terms of diagnostic accuracy compared to 22G FNA needles in focal liver lesions.

Liver imaging provides high diagnostic accuracy in cases of liver lesions, but differential diagnosis might be difficult in cases of a well-differentiated hepatocellular neoplasm or a regenerative nodule, which demand histology. Tissue acquisition can be obtained through ultrasound or CT-guided percutaneous biopsy or through EUS-guided biopsy.

The use of FNA of FNB needles during EUS depends on the choice of endoscopists, pathologists, and, in some cases, the quantity of samples needed for ancillary tests, such as immunohistochemistry (IHC), especially in cases of poorly differentiated neoplasms or for the establishment of the origin of a metastatic focal liver mass. Goldhoff et al. [21] demonstrated that in the case of percutaneous biopsies using FNA needles, the core obtained was more fragmented than in the case of FNB needles, with less non-tumoral tissue, such as stroma and hepatocytes, but with similar cellularity. However, no publication has reported a direct comparison of 22G FNA and FNB needles using the EUS for tissue acquisition.

In the case of diffuse liver disease, a meta-analysis comprising seven studies proved that the length of the specimen when EUS-FNB was used was similar to the percutaneous result [22]. Additionally, 19G FNA needles are preferred over 22G FNB needles due to fragmentation during sample processing, which was interpreted due to specimen friability related to its size [8,23]. Other studies proved that in diffuse liver disease, 19G FNB needles are superior to 19G FNA needles [7] or 22G FNB needles [24].

The diagnostic rate for EUS-FNB was reported to be 93.9%, with an adverse rate of 2.3%, and an insufficient core was considered to be 4% compared to 20% in the case of FNA needles [4]. Furthermore, superior results were achieved with Franseen FNB needles than with Fork-tip needles [25]. Two passes are recommended, ideally from the left lobes of the liver whenever possible [26], although three passes were proven to provide better diagnostic accuracy than one pass, with no supplementary side effects [7].

In the case of focal liver lesions, few articles have been published concerning the needle size or type, with the rate of adverse events varying from 0 to 6.1%, with a median of 3% [27]. The rate of EUS-FNA diagnosis varied between 75 to 100% with two to three needle passes [28], even when an ultrasound contrast agent was used to highlight the lesion [10]. The results of EUS-FNA are comparable to those of percutaneous biopsy, with a lower adverse events rate (2% vs. 17%) [29]. In our study, one pass of FNA provided 86.7% accuracy, similar to the 86.3% accuracy reported by Akay et al. [9] but with a reduced possibility of IHC (histology score of 4 out of 5). We observed no complications, possibly as a result of the restrictive number of passes and the thin 22G needle used. We obtained 100% diagnostic accuracy with Franseen FNB needles, which is comparable to the 97.2% reported in diffuse hepatic diseases [23,30].

First-generation FNB needles have been studied in retrospective studies, showing that the diagnostic accuracy of reverse-beveled needles for solid hepatic lesions was 89.7–90%, and the adequacy for IHC was 88–91% [11,12,13] but without advantages over EUS-FNA needles [14] or 20G antegrade beveled needles (diagnostic accuracy of 88.5%) [31].

A retrospective study including focal liver lesions sampled with multiple types of FNB needles and 19G FNA with multiple passes compared the results to those obtained with percutaneous biopsy, revealing that the EUS-guided technique offered similar adequacy and diagnostic accuracy to the percutaneous technique (88.8% vs. 100% and 92.2% vs. 100%, respectively), with a longer procedure duration (seven vs. one minute). The length of the cores obtained with FNB needles was shorter than that obtained with EUS-FNA (10 vs. 15 mm) [32], which is in contrast to our data (12 vs. 7.9 mm). All histological parameters considered in our study, such as tumor cellularity, core length and surface, and histology score, were superior in the case of EUS-FNB passes relative to EUS-FNA specimens, demonstrating why the adequacy was significantly superior for EUS-FNB samples (90% vs. 63.3%, *p* = 0.01, Table 2).

The strength of our study is associated with the crossover nature of the inclusions and the comparison of the immunohistological quality of the specimens. The limitations of our study include that it is a single-center study and the limited number of patients because of the low number of indications for this procedure. Additionally, all the examinations were performed by an experienced endoscopist, who could not be blinded to the needle type, and the pathologist was very experienced with liver biopsy results. Another limitation is the lack of cytology of the FNA samples, which may have decreased the diagnostic difference, although IHC on cytology specimens is limited. In our analysis of the cores obtained from both EUS-FNA and EUS-FNB, we did not use rapid on-site or macroscopic on site evaluation because we wanted to compare the real value of one pass with EUS-FNA and one pass with EUS-FNB, although their association might improve the accuracy of the EUS-FNA method [17,33,34,35]. We used 20 mL suction during EUS sampling, although further investigation is required for refinement of the technique of tissue acquisition, such as a wet suction technique that minimizes clot formation [36].

## 5. Conclusions

In conclusion, 22G EUS-FNB needles outperform 22G EUS-FNA needles without macroscopic on-site evaluation in terms of diagnostic accuracy and adequacy, with no adverse events, despite higher costs. Further steps are necessary to standardize the technique and the selection of patients, which will aid in the development of this technique of endohepatology.

## Figures and Tables

**Table 1:** Patient characteristics.

Characteristic	Value
Age, years	
mean ± SD	60 ± 12
{min to max}	{37 to 84}
Male sex, n (%)	21 (70.0)
Mass size, cm	
mean ± SD	21 ± 13
Median (Q1 to Q3)	20 (11 to 25)
{min to max}	{6 to 70}
Final diagnosis, n (%)	
Abscesses	6 (20)
Metastasis of pancreatic adenocarcinoma	14 (46.7)
Cholangiocarcinoma	2 (6.7)
Hepatocarcinoma	4 (13.3)
Lymphoma	1 (3.3)
Metastasis of gallbladder carcinoma	3 (10)

**Table 2:** Sample adequacy in the EUS-FNB and EUS-FNA groups and comparisons between FNB and FNA.

	EUS-FNB (n = 30)	EUS-FNA (n = 30)	Stat. (*p*-Value)
Tumor cellularity			
mean ± SD	53 ± 35	39 ± 34	2.9 (0.0039)
Median (Q1 to Q3)	65 (18 to 80)	50 (2 to 68)	
{min to max}	{0 to 95}	{0 to 90}	
Core tissue aggregate length, mm			
mean ± SD	12.1 ± 15.3	7.9 ± 13.7	2.6 (0.0085)
Median (Q1 to Q3)	8 (3.9 to 15.8)	3 (1.1 to 6.8)	
{min to max}	{0 to 71}	{0 to 68}	
Longest core tissue length, mm			
mean ± SD	2.3 ± 1.7	1.4 ± 1.2	2.5 (0.0114)
Median (Q1 to Q3)	2 (1 to 3)	1 (1 to 1.8)	
{min to max}	{0 to 7}	{0 to 4}	
Core surface, mm^2^			
mean ± SD	7.1 ± 9.1	5 ± 8.3	2.4 (0.0179)
Median (Q1 to Q3)	4.6 (2.3 to 8.6)	2.1 (0.7 to 4.8)	
{min to max}	{0 to 42.6}	{0 to 40.8}	
Histology score			
mean ± SD	5 ± 1	4 ± 2	1.4 (0.1730)
Median (Q1 to Q3)	5 (5 to 5)	5 (5 to 5)	
{min to max}	{0 to 5}	{0 to 5}	
Adequate for IHC yield, no./30 (%)	27/30 (90.0)	19/30 (63.3)	6.0 (0.0146)

SD = standard deviation; Q1 = 25th percentile; Q3 = 75th percentile; Stat. = test statistics. FNB and FNA were compared by the Wilcoxon test, except for adequate for IHC (immunohistochemistry), for which the chi-squared test was used.

**Table 3:** Diagnostic metrics in EUS-FNB and EUS-FNA samples.

Metric	EUS-FNB	EUS-FNA
True positives	24	20
True negatives	6	6
False negatives	0	4
False positives	0	0
Sensitivity (%) ^a^	100	83.3 [68.4 to 98.2]
Specificity (%)	100	100
Positive predictive value (%)	100	100
Negative predictive value (%) ^b^	100	60.0 [29.6 to 90.4]
Accuracy (%) ^c^	100	86.7 [74.5 to 98.8]
Negative likelihood		0.17 [0.07 to 0.41]
+CUI	1	0.833 [0.692 to 0.975]
−CUI	1	0.600 [0.374 to 0.826]

^a^ *p*-value = 0.0194; ^b^ *p*-value = 0.0001; ^c^ *p*-value = 0.0387; LB to UP, where LB = lower bound and UP = upper bound of 95% confidence interval.

## Data Availability

The data presented in this study are available in the article.
